# Exploring the impact of frequency of robot’s proactive utterances in product introduction dialogues

**DOI:** 10.1038/s41598-026-49412-3

**Published:** 2026-04-22

**Authors:** Junya Nakanishi, Jun Baba, Yuki Okafuji, Yuichiro Yoshikawa, Hiroshi Ishiguro

**Affiliations:** 1 Engineering Science, The University of Osaka, Osaka, 560-0043 Japan; 2 AI Lab, CyberAgent Inc., Tokyo, 150-0042 Japan

**Keywords:** Human behaviour, Mechanical engineering

## Abstract

This study investigates the impact of the frequency of proactive utterances by interactive agents in product introduction dialogues. Proactive interactive services, unlike traditional reactive services, actively provide information based on user states and situations. This research aims to understand how different frequencies of proactive behaviors affect user evaluation, objective attainment, and the impression of the agent. We developed an interactive agent system capable of delivering proactive utterances at two different frequencies: low and high. The low-frequency condition involved the agent making proactive utterances following its own reactive responses, while the high-frequency condition included additional proactive utterances during silent periods in the dialogue. The experiment compared these two conditions in terms of dialogue duration, information conveyed, user participation, and user impressions. Results indicated that high-frequency proactive utterances led to higher agent proactivity but reduced user proactivity. Moreover, the high-frequency also improved the perceived speaking skills and animacy for the agent. The detailed analysis of high-frequency condition implied that moderate frequency of proactive behaviors are rated higher by users than extremes frequency. These findings highlight the importance of balancing proactive behavior to enhance interactive service experiences without overwhelming the user. Future research should explore these dynamics in more diverse and naturalistic settings to further validate and expand upon these results.

## Introduction

Many studies are conducted on interactive agents such as CG (Computer Graphics) agents and humanoid robots to provide interactive services in public facilities, commercial facilities, educational facilities, and so on^1^. Interactive services are interactions in which an interactive agent provides customer service to a user through words or physical expressions. For example, when a user asks a smartphone, “What’s the weather today?”, a voice assistant provides the weather information. Or, when a user asks a chatbot on an online shopping site, “Tell me more about this product,” the chatbot presents or explains information about the product. As in these examples, interactive services are often “reactive” processes in which the user makes a request via text or voice and the interactive agent responds appropriately. Research is also being conducted to estimate the context of the interaction multimodally to ensure these reactive responses are accurate^2,3^.

In recent years, research on “proactive” interactive services that create opportunities to actively provide interactive services in response to user states and situations without user requests, rather than just passively responding to user requests, has been attracting attention^4–6^. For example, interactive agents predict future behavior based on the user’s past actions or habits, and provide relevant services in advance. Or, when a user arrives at a particular location, interactive agents provide relevant services to that location. In the early studies of proactivity in interactive services, many comparisons were made between proactive and non-proactive (reactive only), and many positive effects (e.g., perceived as more socially appropriate, social, friendly, intelligent, emotional involvement, and warm) and some negative effects (e.g., concerns such as privacy implications, potential loss of agency, and interference with social activities) of proactive behavior were reported^7–16^.

More recently, proactivity has been examined in more detail, broken down along two axes of degree. The first is the degree of “initiative” in carrying out the objectives of the interactive service (which might also be called agency or autonomy). Basically, an interactive service has the goal of satisfying the user (in the case of product introduction, the user understands the product and makes a decision regarding its purchase). The degree of initiative of proactive behavior has been investigated as the degree to which the user controls the actions and decisions toward that objective, or, on the flip side, the degree to which the interactive agent controls actions and decisions to achieve the objective. For example, Kraus et al.^17^ divided the proactive behavior into four levels: the agent performs most of the actions and decisions, the agent suggests actions and decisions, the agent notifies the user that it is able to help, and the agent does nothing (i.e., reactive). All of the studies investigating the degree of initiative commonly report that intermediate degrees of initiative are rated higher than too high or too low initiative in terms of user’s evaluation for agent’s interactive services^17–19^. The second degree of proactivity is “frequency”. Frequency refers to how often the proactive interactive service starts within the possible timing of the services. Buyukgoz et al.^20^ compared three levels of agent’s proactive behavior frequency for cooking recipe generation tasks: high, medium, and none. They report that more frequent proactive behavior results in higher user’s objective attainment but lower user creativity.

While previous studies have provided important insights by comparing proactive and non-proactive agents, most of them focused on the degree of initiative taken by the agent. However, how frequently proactive utterances should occur to optimize user experience remains largely underexplored. Frequency is a distinct dimension from initiative; even agents with the same level of initiative are able to have a difference in how often they start utterances. Therefore, this study pursues the effect of the frequency of proactive interactive services. In particular, this study explores the following three research questions that have been mentioned in previous studies: How does the frequency of proactive behaviors affect the user’s evaluation for interactive services (RQ1), objective attainment (RQ2), and impression of an agent (RQ3)?

As a dialogue scenario, this study focuses on situations in which an interactive agent introduces a product. Product introduction is the process of explaining the features and benefits of a product to a consumer. This process is intended to help the consumer gain a better understanding of the product and get his/her interest or the detailed information to consider purchasing it. Putting an interactive agent in charge of product introduction is a task that has received a lot of attention, as can be seen from the past studies^10,21–26^. The purpose of this study is to investigate the effect of frequency of agent’s proactive utterances in product introduction dialogues. As survey items related to the purpose of the product introduction, we compared the duration of dialogue, the amount of information conveyed about the introduced product, the amount of utterances, the impression of the agent, and the willingness to purchase the product.

For the purpose of this study, we studied the timing of when an interactive service can be actively started in a product introduction dialogue, and developed an interactive agent system that attempts to actively start interactive services with high frequency. Using this system, we conducted an experiment comparing an interactive agent that attempts to start proactive interactive service at two-level frequency (low and high conditions). The low-frequency condition refers to an agent’s strategy of attempting proactive utterances following a its own reactive utterances. The high-frequency condition refers to an agent’s strategy of attempting proactive utterances by detecting a silence period in addition to the low-frequency condition. The results of the experiment suggest the effectiveness and challenges of the proactive interactive services in product introduction situations.

## Results

**Fig. 1:**
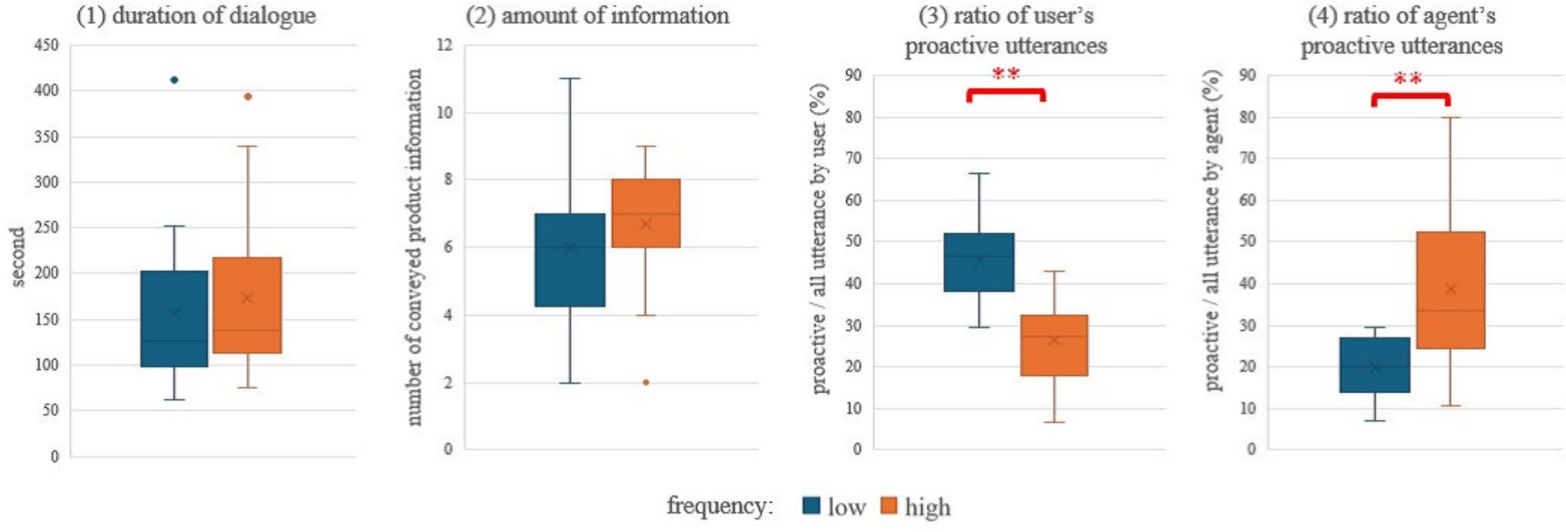
Dialogue status metrics comparing low- and high-frequency conditions. Each box plot shows the median (line), interquartile range (box), outliers (dots), and the mean ($\times$) for the following measures: (1) duration of dialogue (in seconds), (2) amount of conveyed product-related information, (3) ratio of user’s proactive utterances (%), and (4) ratio of agent’s proactive utterances (%). Statistically significant differences (**: $p <.01$) are observed in proactive utterance ratios.

**Fig. 2:**
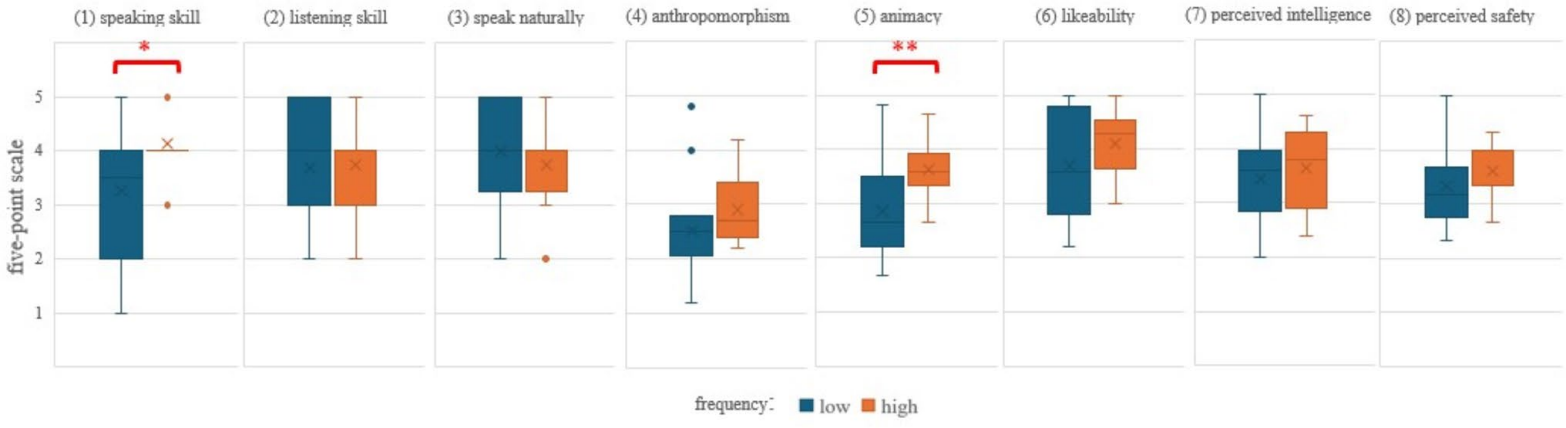
User evaluation scores comparing low- and high-frequency conditions. Each box plot shows the median (line), interquartile range (box), outliers (dots), and the mean ($\times$) for the user evaluation scores. Participants rated the agent’s communicative and social abilities on a five-point scale. Statistically significant differences (*: $p <.05$, **: $p <.01$) are observed in speaking skill and animacy.

The results of analyzing the comparison between low-frequency conditions and high-frequency conditions using a paired-sample t-test and Wilcoxon signed-rank test are described below. A t-test is used for a Likert scale, and a Wilcoxon signed-rank test is used for single items. We applied the Benjamini-Hochberg procedure^27^ to control the False Discovery Rate, which allows for more lenient correction under multiple testing scenarios involving 12 comparisons.

### Dialogue status

Figure 1 shows the duration of the dialogue, the amount of information conveyed about the introduced product, and the ratio of proactive utterances. A significant difference was found in the ratio of proactive utterances (the duration of the dialogue: $Z(15)=0.57$, $p=.60$, $ES(r)=0.14$, the amount of information: $Z(15)=0.85$, $p=.41$, $ES(r)=0.23$, the ratio of user’s proactive utterances: $Z(15)=3.31$, $p=.00021$, $ES(r)=0.83$, the ratio of agent’s proactive utterances: $Z(15)=3.31$, $p=.00021$, $ES(r)=0.83$). The ratio of agent’s proactive utterances is higher in the high-frequency condition than in the low-frequency condition, indicating that the difference in frequency of agent’s proactive utterances between conditions is controlled as planned. On the other hand, the ratio of user’s proactive utterances is lower in the high-frequency condition than in the low-frequency condition.

### User evaluation

Figure 2 shows the results of questionnaires. We found significant differences in robot’s speaking skill and animacy (speaking skill: $Z(15)=2.43$, $p=.015$, $ES(r)=0.81$, listening skill: $Z(15)=0.22$, $p=.85$, $ES(r)=0.067$, speaking naturally: $Z(15)=0.97$, $p=.33$, $ES(r)=0.31$, anthropomorphism: $t(15)=1.42$, $p=.17$, $ES(d)=0.48$, animacy: $t(15)=4.24$, $p=.00072$, $ES(d)=1.06$, likeability: $t(15)=1.89$, $p=.078$, $ES(d)=0.48$, perceived intelligence: $t(15)=0.99$, $p=.34$, $ES(d)=0.24$, perceived safety: $t(15)=1.59$, $p=.13$, $ES(d)=0.45$).

As for willingness to purchase, eight participants answered the high-frequency condition, four answered the low-frequency condition, and four answered no change. The results for each group based on these responses are shown in Table 1.

**Table 1:** The results for each participant group based on willingness to purchase.

	Answered		Answered		Answered	
	HIGH		LOW		NO CHANGE	
	LOW	HIGH	LOW	HIGH	LOW	HIGH
	[DIF]		[DIF]		[DIF]	
Duration of the dialogue	176.69	202.02	141.68	138.94	134.68	149.40
	[+25.33]		[−2.74]		[+14.72]	
Amount of information	5.75	7.00	6.00	6.00	6.50	6.75
	[+1.25]		[0.00]		[+0.25]	
Ratio of user’s proactive	45.70	25.76	48.60	20.81	41.80	33.64
	[−19.94]		[−27.79]		[−8.16]	
Ratio of agent’s proactive	20.55	38.06	19.55	52.38	19.22	27.06
	[+17.51]		[+32.83]		[+7.84]	
Speaking skill	2.50	4.00	4.00	4.25	4.00	4.25
	[+1.50]		[+0.25]		[+0.25]	
Listening skill	3.50	3.50	4.25	3.75	3.50	4.25
	[0.00]		[−0.50]		[+0.75]	
Speaking naturally	3.63	3.38	4.75	3.75	4.00	4.50
	[−0.25]		[−1.00]		[+0.50]	
Anthropomorphism	2.10	2.88	3.55	3.10	2.35	2.75
	[+0.78]		[−0.45]		[+0.40]	
Animacy	2.31	3.54	3.71	4.04	3.08	3.38
	[+1.23]		[+0.33]		[+0.29]	
Likeability	3.18	4.00	4.45	4.30	4.00	4.10
	[0.83]		[−0.15]		[+0.10]	
Perceived intelligence	3.10	3.73	4.30	3.60	3.30	3.50
	[+0.63]		[−0.70]		[0.20]	
Perceived safety	2.96	3.58	4.17	3.75	3.25	3.50
	[+0.63]		[−0.42]		[+0.25]	

LOW and HIGH indicate a frequency condition.

Values in [DIF] indicate the increase from LOW to HIGH.

## Discussion

### Manipulation of dialogue status

In the high-frequency condition, the ratio of agent’s proactive utterances was significantly higher (Fig. 1). The agent in the high-frequency condition was able to make proactive utterances even if the user did not respond, so it is likely that the ratio of agent’s proactive utterances could have been higher. This indicates that the frequency of the agent’s proactive utterances in the dialogue was controlled in the high-frequency and low-frequency conditions, as assumed in the experimental design. In addition, the ratio of the user’s proactive utterances is decreasing as the agent’s proactive utterances increase. The ratio of the user’s proactive utterances seems to be inversely proportional to the agent’s proactive utterances.

### Evaluation for interactive service

For the response to willingness to purchase, descriptive trends in participants’ responses suggest that the high-frequency condition may have been more persuasive in some cases. However, as this measure was not subjected to statistical testing, this finding should be regarded as preliminary and hypothesis-generating rather than conclusive. Below, we will discuss the details of each response group (Table 1).

First, participants who endorsed the low-frequency condition showed the highest ratio of increase in the ratio of agent’s proactive utterances and the highest ratio of decrease in the ratio of user’s proactive utterances in the high-frequency condition compared to the low-frequency condition. As a result, the ratio of agent’s proactive utterances was the highest and the ratio of user’s proactive utterances was the lowest in the high frequency condition. This means that the participants who endorsed the low-frequency condition handed over the initiative of the dialogue to the agent most in the high-frequency condition. In addition, participants showed the least positive change between conditions in the duration of the dialogue and the amount of information conveyed about the introduced product, suggesting that they were relatively quick to cut off the dialogue despite the agent’s proactive utterances. In the interviews, the participants complained about the agent’s one-way explanation of the product (three of four participants). This may be because the high-frequency condition was too proactive for those participants to be able to talk, and they lost motivation to engage in the product introduction dialogue. As a cause of this, the experimenter’s impression from observation was that those participants seemed to pause in silence between their utterances long enough to be interrupted by the agent’s proactive utterances.

Conversely, participants who did not endorse differences between conditions (i.e., no change) showed the least change in the ratio of agent’s and user’s proactive utterances. This means that there was no significant difference in the initiative of the dialogue between conditions, suggesting that the participants were unable to recognize the difference. As a cause of this, the experimenter’s impression from observation was that those participants seemed to be speaking incessantly, to the point that the agent was unable to make proactive utterances.

The participants who endorsed the high-frequency condition were in between those who endorsed the low-frequency condition and those who indicated that there was no difference between the conditions in terms of changes in the ratio of agent’s and user’s proactive utterances. The duration of dialogue and the amount of information conveyed about the introduced product were highest in the high-frequency condition.

The above trends suggest a very important hypothesis in the adjustment of frequency. That is, the frequency of the agent’s proactive utterances should be moderate, neither too much nor too little. As mentioned above, if the agent actively engages in proactive utterances to the extent that it causes the user to refrain from speaking, the user will be dissatisfied with not being able to participate in the dialogue, and his/her willingness to engage in the dialogue will seem to decrease. On the other hand, if the agent’s proactive utterances are so modest in frequency that the user does not perceive any change in the dialogue, the user will not perceive any difference. It seems that proactive utterances with just the right frequency that does not frustrate the user are required. Furthermore, there is variation (i.e., individuality) in the way users pause between utterances, suggesting that it is important to adjust timing of proactive utterances accordingly.

The hypothesis that user evaluation of moderate frequency (neither too much nor too little) in terms of an interactive service are higher is consistent with previous research and extends the findings. Studies on the degree of initiative of proactive behaviors commonly report that intermediate initiative is rated higher than too much or too little initiative in terms of user’s evaluation for agent’s interactive services^17–19^. Peng et al.^18^ explains that high initiative increases the likelihood that the user will judge it as inappropriate. The results of this experiment suggest a similar trend in the frequency of proactive behaviors. A frequency that is not stressful to the user is considered important.

### Evaluation for objective attainment

No significant changes were observed in the duration of the dialogue or the amount of information conveyed about the introduced product. In the high-frequency condition, the agent can start proactive utterances by detecting the silent periods of the dialogue, which would be expected to make the dialogue last longer and increase the amount of information that could be provided, but this was not the case. One possible reason for this may be that the user is able to terminate the dialogue at his/her own will. Whether the user asks questions to obtain information or the agent proactively provides information, it is possible that the user is satisfied with the dialogue and leaves the product introduction dialogue with the agent when a certain dialogue time or a certain amount of information is reached. The fact that the ratio of user’s proactive utterances and the ratio of agent’s proactive utterances appear to be inversely proportional supports this assertion. Simply responding to the user’s needs ahead of time may only make the difference between user- and agent-oriented processing of the user’s needs, and may have little effect on the dialogue time or the amount of information exchanged.

### Impression of agent

The results of this experiment suggest that the effect of high-frequency proactive behaviors on impressions of the agent is an improvement in speaking skills and animacy. In terms of speaking skills, the most common opinion in the interviews (seven participants) was that participants appreciated that the agent start to speak without the they having to ask questions in the high-frequency condition. In terms of animacy, the most common opinion in the interviews (eight participants) was that they felt that the agent was not biological in that it was not able to respond unless the they asked it a question in the low-frequency condition.

Comparative studies with and without proactive behaviors reported, for example, that proactive behaviors improved social appropriateness in the product introduction dialogue^10^ and naturalness, friendliness, and intelligence in the reception task^11^. It is important to note that the specific aspects of the impression that change can vary depending on the nature of the proactive behaviors and the context of the task. Future research should explore these nuances further to better understand the mechanisms behind these impression changes and to identify optimal strategies for utilizing proactive behaviors in various interactive contexts.

### Limitations and future work

This study has several limitations that should be acknowledged.

First, the sample size was relatively small, comprising 16 participants, all of whom were university students or staff in their 20s and 30s. While this group allowed us to control demographic variables and maintain internal consistency, it may not represent the broader population in terms of age, occupation, or digital literacy. As such, the statistical power of the study is limited, and caution should be exercised when generalizing the findings to more diverse populations. Future studies with a larger and more varied sample would enhance the robustness and reliability of the results.

Second, all participants were native Japanese speakers, and the interaction was conducted in Japanese. Therefore, the results may be culturally and linguistically specific, and further research is needed to determine whether similar effects occur in different cultural or linguistic contexts.

Third, the experiment was conducted in a controlled laboratory environment, which provided consistency in experimental conditions but may not reflect the complexity and variability of real-world settings in which interactive agents are typically used. Participants were asked to imagine themselves in a specific context (i.e., at an airport souvenir shop), but this imaginative exercise may not fully replicate the spontaneity and behavioral dynamics of real-life situations.

Given these limitations, we suggest that future work include field-based experiments in naturalistic environments, such as retail stores or public information kiosks, to validate the findings in more ecologically valid conditions. Such studies would help assess how proactive utterance frequency influences user experience in everyday human-agent interactions and improve the generalizability of the results.

## Methods

In this section, we organize the situation of being in a dialogue and discuss the timing of utterances that can start proactive interactive services. We then design a method to investigate how varying frequencies of proactive utterances by robots affect user perception, conversational dynamics, and overall impression. This research is based on sophisticated experimental methods in human-robot interaction^28,29^.

### Proposed technique

#### Theory and design concept

Dialogue consists of utterances (and equivalent actions) by each interlocutor. An utterance is an act of communicating to the other interlocutors by speaking (or by other means). Here, however, we distinguish backchannels (e.g., “Uh-huh” and “Yeah”) from utterances and do not include it in utterances. Since it is difficult for a human in a dialogue to process both speaking and receiving the other’s speech at the same time, the dialogue is basically conducted so that interlocutors do not speak at the same time. However, if each interlocutor starts speaking at will without any countermeasures, speech collisions can easily occur. For this reason, rules regarding a “speech turn”, which are the recognition of which interlocutor is allowed to speak at this time, are shared, and a speech collision is avoided by switching speakers (called “turn-taking”^30^). For example, if an interlocutor utters filler words such as “Well...” or “Um...”, it is an indication that the interlocutor intends to start speaking, and the interlocutor’s speech turn often starts. If an interlocutor makes a questioning utterance and then shows an attitude of waiting without continuing the speech, it is regarded as an act of passing the speech turn to the other interlocutor. Under such a rule, interlocutors have a common understanding of who holds the speech turn, and only the holder of the speech turn can speak, thus avoiding collisions in dialogue . Based on these rules, it can be said that there are user’s and agent’s speech turns in a user–agent dialogue, and agent’s proactive utterances should be avoided during the user’s speech turn.

During the agent’s speech turn, it is a permissible time for the agent to speak, but it is not necessarily a permissible time to start a proactive interactive service. There are two types of agent’s speech turn: those that a user seeks a response, such as request utterances in reactive interactive services, and those that a user does not seek a response. The response-seeking utterance includes questions, part of commands, part of greetings, etc., while the non-response-seeking utterance includes explanations, agreements, apologies, etc., if we use the classification concept of dialogue act^31^ as an example. Agent’s speech turn after a response-seeking utterance is one that makes the user feel ignored if no response is given in response to the utterance. The service provider, the agent, wants to avoid giving the user, the service user, an unpleasant feeling such as being ignored, because it may lead to a decrease in service satisfaction. Therefore, it can be said that when the agent’s speech turn caused by the user’s response-seeking utterance, the agent should start with an utterance in response to the user’s seeking for response (i.e., reactive interactive service), and proactive interactive service concerning a different topic should be avoided.

On the other hand, it is pointed out that the holder of a speech turn is sometimes clear and often ambiguous^32^. For example, after response-seeking utterance, the holder of the speech turn is clear in most cases, but after non-response-seeking utterance, the holder is ambiguous. It would not be unnatural if the speaker himself who has made the non-response-seeking utterance continues to speak, or if his listener makes the utterance. Hence, when the holder is ambiguous, the agent is allowed to start a proactive interactive service. The timing when the speech turn holder is ambiguous is after a non-response-seeking, or after a response-seeking but the responder does not speak.

In summary, the above discussion suggests that proactive interactive service can start (1) after user’s non-response-seeking utterance, (2) after agent’s response-seeking utterance but no response from a user, and (3) (4) after agent’s non-response-seeking utterance (Table 2).

**Table 2:** When it is possible to start proactive interactive service?

Dialogue situation	permissible or impermissible?
After user’s response-seeking utterance	Impermissible; because agent should respond user’s seeking for response
After user’s non-response-seeking utterance	Permissible (1)
After agent’s response-seeking utterance	Impermissible; because during user’s speech turn or permissible if no response from a user (2)
After agent’s non-response-seeking utterance	Permissible (3) or permissible if no response from a user (4)

#### System architecture and implementation

Based on the discussion in the previous section, this section describes an agent system that can start proactive as well as reactive interactive services. Since the reactive interactive service is intended to respond to user’s seeking for response, it can start from the detection of the end of the user’s response-seeking utterance. The detection of the end of the user’s request utterance is determined by the detection of the end of the user’s utterance and the detection of the fact that the user’s utterance is a response-seeking utterance. Proactive interactive services during a dialogue can start in (1) to (4) of Table 2. The detection of the end of the user’s non-response-seeking utterance (1) is determined by the detection of the end of the user’s utterance and the detection that the user’s utterance content is not a response-seeking utterance. Detection of the end of an agent’s response-seeking utterance but no response from the user (2) is judged by detection of the end of the agent’s response-seeking utterance and detection of a certain silence interval thereafter. The end of the agent’s non-response-seeking utterance is assumed to have two patterns: immediately after the end of the agent’s non-response-seeking utterance (3) and after a certain silence interval (4). In the case of immediately after, the end of the agent’s non-response-seeking utterance need not be detected. When the agent generates a non-response-seeking utterance, it can simultaneously generate a proactive utterance. For example, agents can consider a proactive utterance simultaneously in the generation of non-response utterance in response to the user’s response-seeking utterance, and in a form that follows that utterance. The detection of a certain silence interval is determined by the detection of the end of the agent’s non-response-seeking utterance and the detection of a certain silent interval afterwards.

**Fig. 3:**
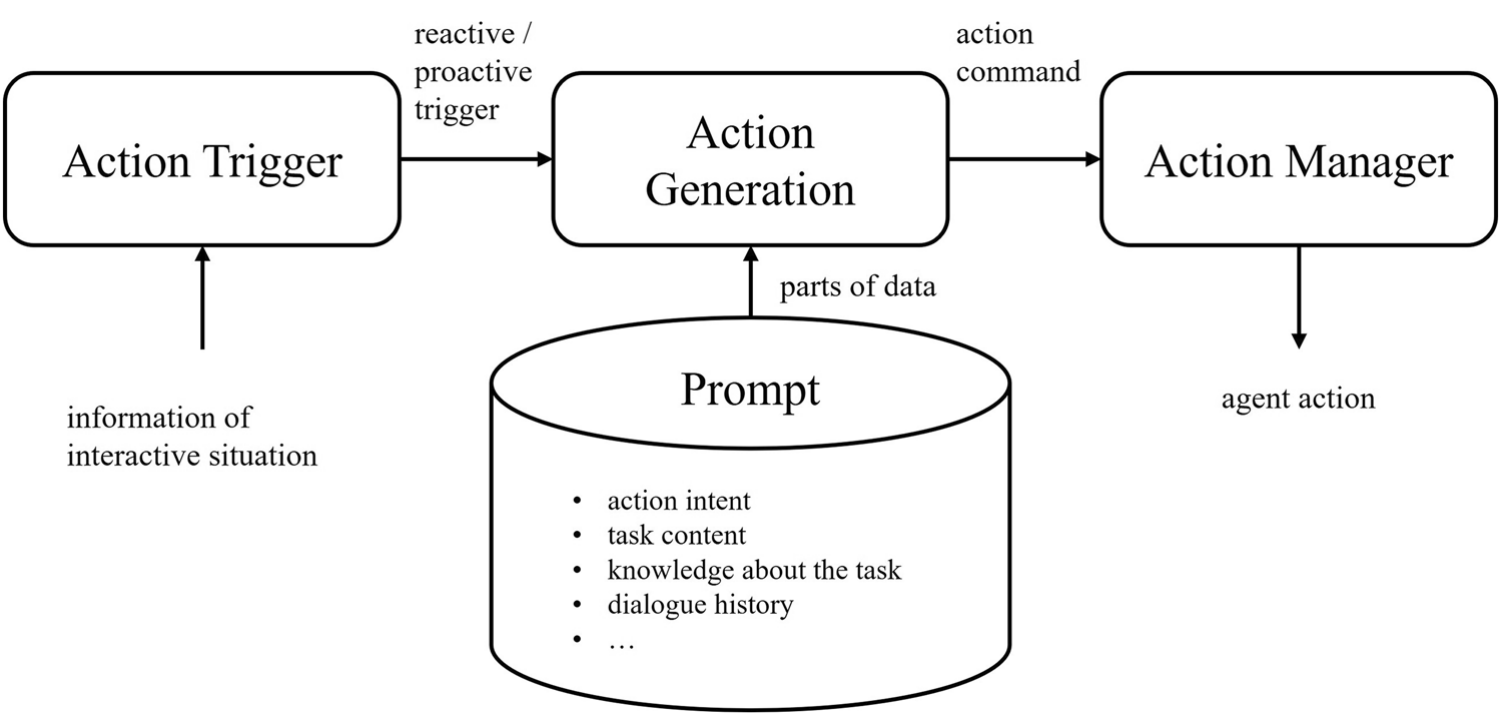
The system architecture.

By implementing the above algorithm for speech start as the Action Trigger module and combining it with the Action Generation module and the Action Manager module, we implemented an interactive agent system that can also provide proactive interactive services. Figure 3 shows an overview of the architecture of this system. The Action Trigger is a module that outputs the start timing of action and its action intention (proactive or reactive in this study) based on the information of the interactive situation as input, and passes the output results to the Action Generation. The Action Generation takes information about action (e.g., action intent, task content, knowledge about the task, and dialogue history) as input, outputs the agent’s action content, and passes it to the Action Manager. The Action Manager stores the received action contents and outputs them as actual actions in a queued format or cancels them, while adjusting the timing according to the information on the dialogue status. This Action Manager plays an equally important role as the Action Trigger module in managing the timing of utterances. For example, it waits for the output of utterances until the end of the user’s speech turn, or swaps the waiting utterances with newly generated ones, to achieve an adaptive response while following turn-taking.

The experimental implementation uses WebRTC (Web Real-Time Communication, Google LLC) for recognition of the user’s speech turn, GPT-4TURBO (OpenAI Inc.) for speech content generation, Sota robot (Vstone Co., Ltd.) and its control system as the interactive agent. For the detection of silent intervals, 2 sec was used based on previous studies^33,34^. The robot’s control system was based on the experimenter’s previous research and implemented autonomous breathing behavior and representation of body movements associated with speech^35^.

### User study

#### Study design

Conventional reactive interactive agents, which respond only to the user’s utterances, can only consider providing proactive interactive services in the opportunities in (1) and (3) of Table 2. In this study, we have found additional opportunities to provide proactive interactive services in (2) and (4) of Table 2. Hence, we investigated the impact of these additions on dialogue. In other words, we conducted a comparative experiment between a low-frequency condition, which is proactive interactive service for (1) and (3), and a high-frequency condition, which is proactive interactive service for (1) to (4).

- **Low-frequency condition** Proactive utterances are performed as well as reactive utterances in the way of response to the user.
- **High-frequency condition** Proactive utterances are performed by detecting silent pose in addition to the low-frequency condition.

**Fig. 4:**
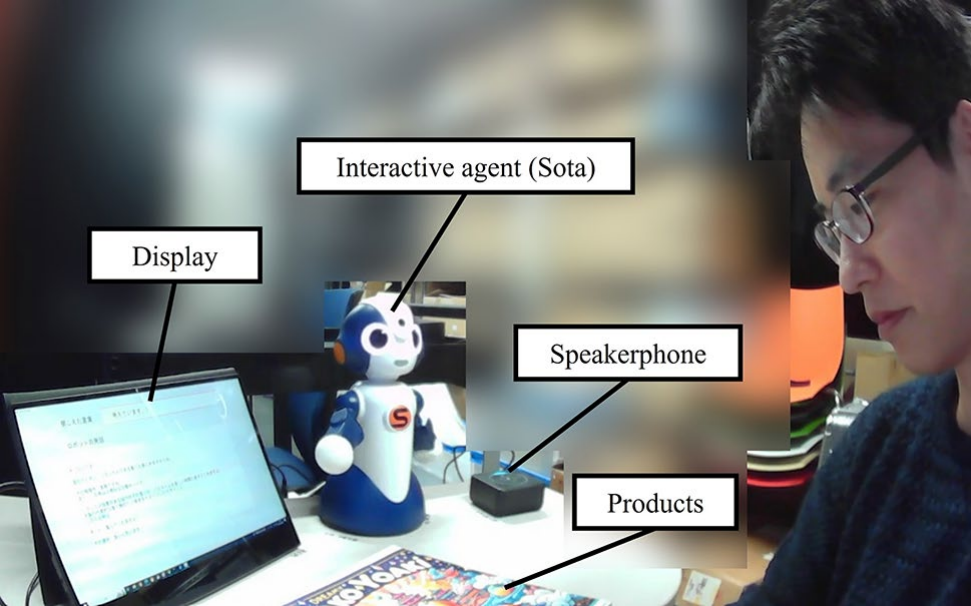
The experimental setup.

We designed a within-subject experiment in which each subject experiences both agents in the two conditions. The intention of employing a within-subjects experiment rather than a between-subjects experiment was to elicit from the subjects the differences between the conditions. The significance level was set at 5%. This study was approved by the ethics committee of the Graduate School of Engineering Science at Osaka University and the methods were performed in accordance with the approved guidelines. Also, we obtained informed consent from the participant and the experimenter (Fig. 4) to publish their images.

#### Scope of inquiry

In this study, we focused on situations in which an interactive agent introduces a product as one of the interactive service situations. Product introduction is the process of explaining the features and benefits of a product to a consumer. This process is intended to help the consumer gain a better understanding of the product to consider purchasing it. This study explores the impact of frequency of proactive behaviors on the user’s evaluation for interactive services (RQ1), objective attainment (RQ2), and impressions of an agent (RQ3). Items related to these RQs were the scope of the inquiry. First, as survey items related to the user’s evaluation for interactive services, we measured the willingness to purchase it. As survey items related to user’s objective attainment, we compared the amount of information conveyed about the introduced product and the duration of dialogue. As survey items related to user’s impressions of an agent, we compared communication skills and concepts relevant to Human–Robot Interaction.

#### Participants

A total of 16 participants (6 women and 10 men, students and staff in their 20s and 30s) participated in this experiment. The number of 32 data taken from 16 participants was an adequate sample size to detect a large-level effect size in a paired-sample t-test (calculated by G*Power^36^: effect size *d* = 0.75, significance level = 0.05, power = 0.8) and a Wilcoxon signed-rank test (effect size *d* = 0.77, significance level = 0.05, power = 0.8). We note that, consistent with the exploratory nature of the study, the power analysis was not intended to provide a precise a priori estimate of the true effect size, but rather to ensure that the design would be sensitive enough to capture robust, large effects should they occur. All participants were involved in research work at the university. They had never been involved in this study, although the Sota robot was not their first. They are native Japanese speakers and interacted with the robot in Japanese. All participants consented and signed a form giving permission to use and share anonymous data for scientific purposes.

#### Procedure

The procedure for all participants was identical except for the order of the experimental conditions. We used a counterbalanced design to evenly distribute any potential order effects across conditions. After signing the informed consent form, participants were briefed on the experimental procedures, but were not given any information about the experimental conditions. Participants were told to assume a situation in which they were at the airport on their way home from a trip to Osaka and were stopping at a souvenir shop. There, they were told to imagine a situation in which they found a souvenir corner where a robot was installed and stopped by. They were informed that there was no time limit set for the experimental setting, and they were told to interact with the robot until they were satisfied. After the dialogue, participants completed a questionnaire on a web form. At the start of the second condition, participants were instructed to forget their memories of the first experience. At the end of the two dialogue-evaluation cycles, an oral interview was conducted.

#### Setup

The experiment was conducted in a laboratory at the university. The experimental setup is shown in Fig. 4. Participants sat in front of a robotic system and received interactive services. The participant was able to view the product samples and participate in the product introduction dialogue. The robotic system consisted of a Sota robot and a display, and the display showed the participant’s speech as recognized by the system and the robot’s speech. This was provided to allow for visual confirmation of any discrepancies in speech recognition between each other. The product was a fictitious product associated with Osaka. The product idea, product name, detailed information about the product, and product packaging were based on the GPT-4 output.

Two types of prompts for generating speech content (GPT-4TURBO) were prepared: one for responding to user’s utterances and the other for silent sections. The prompts for responding to the user’s utterances instructed to respond to the user’s requests and demands as reactive behavior, and to provide product information that has not been conveyed and to ask about the user’s needs as proactive behavior, which realizes (1) and (3) of Table 2. In the prompts for the silent section, only instructions for proactive behavior were used, which realizes (2) and (4) of Table 2. The silence section response can only be triggered twice in a row at most. The common prompts include a description of the task of introducing the product and information about the product.

#### Measurement

The amount of information conveyed about the introduced product, the duration of the dialogue, and the ratio of proactive utterances were measured by one annotator from the video data of the dialogue, and the validity of all annotations was checked with the experimenter. Through this procedure, complete agreement was reached for all measurements. Although inter-rater reliability scores were not calculated, the annotation followed consistent criteria and was cross-validated to ensure accuracy and consistency.

The amount of information conveyed about the introduced product: it was counted among the 16 pieces of product information observed during the entire experimental dialogue. For example, price, expiration date, shape of the product, and other customers’ evaluations. The duration of the dialogue: it was measured from the point of dialogue starting in the video data until the user left the dialogue.

The ratio of proactive utterances: firstly, the amount of utterance was counted as one unit of consecutive utterances without a silence interval of 2 sec or longer in the utterances excluding the backchannels. There are two reasons for adopting this threshold (2 sec): (1) this standard used in this work with robots to determine the timing of additional utterances, and (2) it is based on conversational norms: humans typically begin speaking within 2 sec if they have something to say^37^. After counting, each user’s and agent’s utterance was labeled as proactive or reactive.

The agent’s dialogue skill and impression of the agent were measured by questionnaire. A five-point scale was used for each question item. The following three questions^38^ were used to measure the agents’ dialogue skill: “Do you think the agent’s speaking skills are good?”, “Do you think the agent’s listening skills are good?”, “Do you think the agent was speaking naturally?”. Godspeed^39^ was used to measure the agents’ impressions of the agents. The Godspeed Questionnaire Series is widely used in human-robot interaction research to assess users’ perceptions of robots. The Cronbach’s coefficients for each dimension are as follows: anthropomorphism:$\alpha =.80$, animacy:$\alpha =.84$, likeability:$\alpha =.91$, perceived intelligence:$\alpha =.87$, perceived safety:$\alpha =.13$. Since internal consistency could not be confirmed only for safety, it was not included in the discussion.

Finally, participants were asked which of the two conditions was higher in the interviews regarding their willingness to purchase (the first condition, the second condition, or no change). Also during the interviews, participants were asked about the reasons for the differences between conditions in each of the items in the questionnaire.

## Data Availability

The datasets used and/or analysed during the current study available from the corresponding author on reasonable request.
